# Surgery or Comorbidities: What Is the Primum Movens of Kidney Dysfunction After Nephrectomy? A Multicenter Study in Living Donors and Cancer Patients

**DOI:** 10.3390/jcm13216551

**Published:** 2024-10-31

**Authors:** Francesco Trevisani, Matteo Floris, Francesco Trepiccione, Giuseppe Rosiello, Giovambattista Capasso, Antonello Pani, Marco Maculan, Giacomo Mascia, Cristina Silvestre, Arianna Bettiga, Alessandra Cinque, Umberto Capitanio, Alessandro Larcher, Alberto Briganti, Andrea Salonia, Paolo Rigotti, Francesco Montorsi, Andrea Angioi, Lucrezia Furian

**Affiliations:** 1 Division of Experimental Oncology, Urological Research Institute, IRCCS San Raffaele Scientific Institute, 20132 Milan, Italy; rosiello.giuseppe@hsr.it (G.R.); bettiga.arianna@hsr.it (A.B.); capitanio.umberto@hsr.it (U.C.); larcher.alessandro@hsr.it (A.L.); briganti.alberto@hsr.it (A.B.); salonia.andrea@hsr.it (A.S.); rigotti.paolo@hsr.it (P.R.); montorsi.francesco@hsr.it (F.M.); 2 Department of Urology, IRCCS San Raffaele Scientific Institute, Vita-Salute San Raffaele University, 20132 Milan, Italy; 3 Biorek srl, San Raffaele Scientific Institute, 20132 Milan, Italy; alessandra.cinque@biorek.eu; 4 Department of Nephrology, Dialysis, and Transplantation, G. Brotzu Hospital, 09134 Cagliari, Italy; matteo.floris@aob.it (M.F.); antonellopani@aob.it (A.P.); giacomo.mascia@aob.it (G.M.); andrea.angioi@aob.it (A.A.); 5 Department of Translational Medical Sciences, University of Campania “Luigi Vanvitelli”, 80138 Naples, Italy; francesco.trepiccione@unicampania.it (F.T.); gb.capasso@unicampania.it (G.C.); 6 Kidney and Pancreas Transplantation Unit, Department of Surgical, Oncological and Gastroenterological Sciences, University of Padova, 35122 Padova, Italy; marco.maculan@unipd.it (M.M.); cristina.silvestre@unipd.it (C.S.); lucrezia.furian@unipd.it (L.F.)

**Keywords:** AKI, CKD, living kidney donor, renal cancer

## Abstract

**Background and Hypothesis**: Acute Kidney Injury (AKI) and Chronic Kidney Disease (CKD) are significant risks for kidney cancer (KC) patients undergoing partial (PN) or radical nephrectomy (RN) and for living kidney donors (LKD). This study compares AKI and CKD incidence in these groups with a pre-operative glomerular filtration rate (GFR) over 60 mL/min/1.73 m^2^. **Methods**: This study included 465 KC patients with cT1-2N0M0 kidney mass and 256 LKD who underwent nephrectomy at four Italian institutions from 2014 to 2021. Data on demographics, comorbidities, and therapies were analyzed. Serum creatinine and estimated GFR (eGFR) were measured before and after surgery. Outcomes were AKI (per KDIGO guidelines) and CKD stage progression. Analyses included descriptive statistics, ANOVA, logistic regression, and Kaplan–Meier survival. **Results**: Among 721 patients, significant age and gender differences were noted. Hypertension (41%) and diabetes (7.1%) were prevalent in RN and PN groups. Post-surgery AKI was more common in donors (84%), while CKD stage progression varied by surgery type (CKD stage G3 after 60 months: RN 48.91%, PN 18.22%, LKD 26.56%). Age, pre-surgery CKD, and surgery type predicted CKD progression. Limitations include retrospective design and bias. **Conclusions**: Both LKD and KC patients face similar AKI and CKD risks. Surgery type significantly influences AKI and CKD incidence, highlighting the importance of approach.

## 1. Introduction

Kidney surgery represents one of the most critical risk factors for developing acute kidney injury (AKI) and chronic kidney disease (CKD) [1,2]. After surgery, the loss of nephron mass leads to compensatory hyperfiltration, which may result in maladaptive mechanisms that paradoxically enhance the loss of nephrons over time [3,4]. Since the peri-operative onset of a mild to severe degree of AKI is a widely known predictive risk factor of poor kidney outcomes (as CKD of any degree to End Stage Kidney Disease (ESKD)), it is essential to find reliable markers to stratify candidates and intercept those at higher risk accurately [5,6].

Previously published data on patients with kidney cancer (KC) showed that the type of surgery (partial nephrectomy PN vs. radical nephrectomy RN) and the surgical technique used (open, laparoscopic, or robotic), along with pre-existing comorbidities such as diabetes, CKD, hypertension, obesity, and cardiovascular diseases, could help identify which patients are at a higher risk of developing AKI or CKD, or both, at the baseline [5,6,7].

However, despite the many reports regarding KC in the literature, few studies have elucidated these mechanisms well in another clinical setting, the living kidney donors (LKD) [8,9].

Kidney transplant is the best replacement therapy, and LKD is an increasing supply of organs for ESKD patients to stop or avoid dialysis. To ensure optimal outcomes after donation, LKD must be carefully monitored by clinicians to prevent AKI and CKD over time [10]. It is common knowledge that KC patients, although with a normal kidney function at baseline, may experience a worse kidney functional impairment in comparison to LKD because KC patients are usually more fragile than their healthy counterparts [11,12]. By including both LKD and kidney cancer patients undergoing different surgical procedures, we aimed to isolate the effects of surgery type (extent of nephron loss) from patient-related factors (presence of comorbidities like cancer).

But are we utterly confident in this dogma?

Our study investigated whether LKD and KC patients with normal kidney function at baseline display different outcomes in acute and chronic settings. It evaluated the onset of AKI and CKD in a cohort of 721 patients enrolled in four tertiary Institutions.

## 2. Materials and Methods

### 2.1. Study Population

A total of 465 consecutive patients who underwent either PN or RN for cT1-2N0M0 kidney mass and 256 patients who underwent nephrectomy as LKD at four Italian institutions (IRCCS San Raffaele Hospital, Milan; AOU Padova; ARNAS “G. Brotzu”, Cagliari; AOU Policlinico Universitario “L. Vanvitelli”, Napoli) between 2014 to 2021 were included.

To be eligible for the study, participants had to meet the following criteria: (1) be at least 18 years old, (2) have no history of kidney failure before surgery, as defined by KDIGO 2012 guidelines (eGFR ≥ 60 mL/min/1.73 m^2^ using CKD-EPI 2021 formula and no urinary abnormalities), (3) have no metastatic disease or be receiving concurrent tyrosine kinase inhibitors (TKI) and/or immunotherapy, and (4) have signed an informed consent form.

### 2.2. Covariates and Outcomes

Cofounders were age, gender, boy mass index (BMI), grading, hypertension, diabetes, and medical therapy (e.g., ACE inhibitors (ACEis), angiotensin II receptor blockers (ARBs), calcium channel blockers, beta-blockers, and diuretics). The serum creatinine (sCr) and eGFR (CKD-EPI formula) were measured at predefined time points during follow-up (Time 0, 24, 48, and 72 h after surgery, at discharge, and 6, 12, 24, 36, 48, and 60 months after surgery).

Two primary endpoints were considered: (1) the development of AKI (any degree) and (2) CKD stage progression or appearance. AKI was defined and stratified based on the KDIGO 2012 Clinical Practice Guideline for Acute Kidney Injury. We stratified CKD classes according to the KDIGO 2012 Clinical Practice Guideline for the Evaluation and Management of Chronic Kidney Disease [13].

### 2.3. Statistical Analyses

The study used descriptive statistics to summarize the data. Normally distributed variables were described as mean and standard deviation (±SD), while median and interquartile range were used if data deviated from the standard curve. Categorical variables were expressed as absolute frequencies and percentages.

A comparison between the considered groups (RN vs. PN vs. LKD) and outcomes was performed using one-way ANOVA for categorical variables; a post hoc analysis was conducted with Fisher’s Least Significant Difference (LSD) method to determine which specific pairs of groups differ significantly from each other. The relationship between the covariates (continuous and dichotomous variables) and the considered outcomes (development of AKI, change of CKD class) was investigated using logistic regression.

Survival analysis was conducted using Kaplan–Meier curves, and then the results were compared using the Mantel–Cox Log-rank test. We considered a *p*-value less than or equal to 0.05 as statistically significant.

To assess the independent effects of covariates on the likelihood of outcomes, we employed a logistic regression model as univariate and multivariate analysis. We chose the “Enter” method for variable inclusion, which means all covariates of interest were simultaneously included in the multivariate model.

Statistical analysis and database management were conducted using IBM^®^ SPSS^®^ Statistics Version 29 (IBM Corp., Armonk, NY, USA).

## 3. Results

A total of 721 patients were included in the study based on the inclusion criteria. Out of these, 465 patients (64.4%) underwent either an RN (49.2%) or PN (50.8%). The remaining 256 patients (35%) were LKD (as shown in Table 1). The ages of the patients who underwent RN and PN were similar, with an average of 56.9 years (±10.83) and 57.18 years (±10.74), respectively. However, the age of kidney donors was comparatively younger, with an average of 50.8 years (±9.9).

Among the patients who underwent RN or PN, 68.8% were male. Conversely, 28.5% of the kidney donors were male. Most of the patients were overweight, with an average BMI of 26.16 (±4.5), except for the kidney donors, who had a comparatively lower BMI of 24.7 (±3.25).

**Figure 1:**
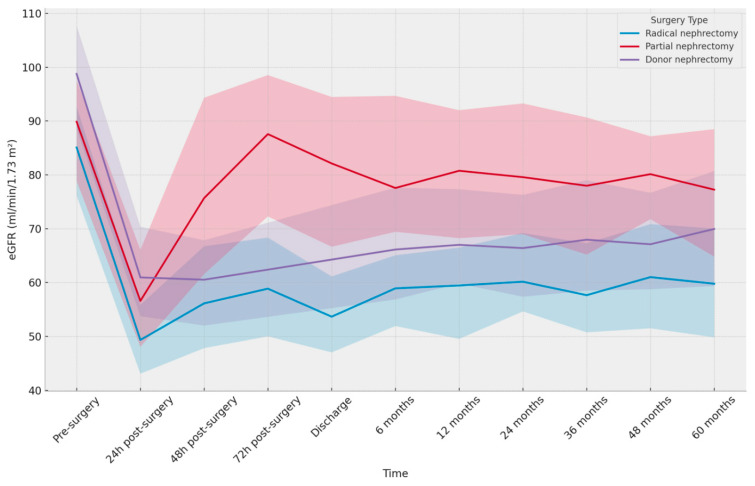
Median eGFR over time for each surgery type (with extrapolation). Line plot with extrapolated median eGFR values and their corresponding interquartile range (IQR) according to the surgery type (Radical nephrectomy, Partial nephrectomy, and Donor nephrectomy). The x-axis represents time, ranging from pre-surgery to 60 months post-surgery. The y-axis represents eGFR, measured in mL/min/1.73 m^2^. The shaded area around each line represents the IQR (25th to 75th percentile) for the corresponding surgery type at each time point.

Patients who underwent RN or PN had a higher incidence of conditions like hypertension (41%), type 2 diabetes (7.1%), and a history of smoke (43.2%). Before the procedure, both RN and PN patients had similar kidney function values (eGFR CKD-EPI: 87.2 ± 11.6 mL/min/1.73 m^2^ and 91.1 ± 12.5 mL/min/1.73 m^2^, respectively). On the other hand, LKD patients had significantly higher values (eGFR CKD-EPI: 97.40 ± 13.27 mL/min/1.73 m^2^) (Figure 1).

**Figure 2:**
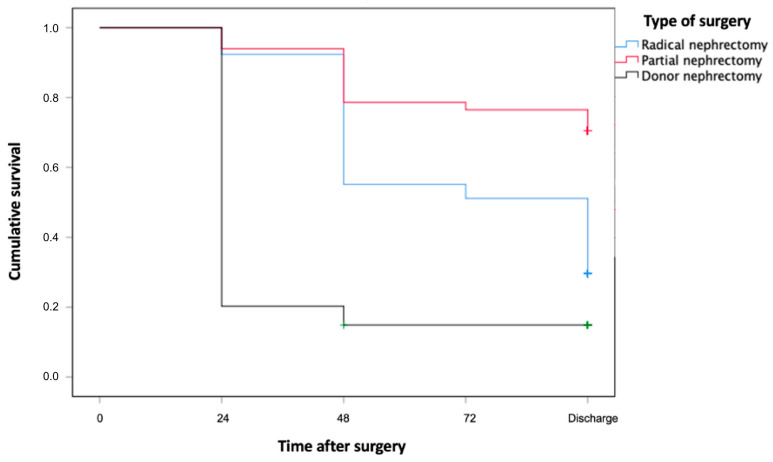
Survival analysis between the surgery groups at time 0 (pre-surgery) and discharge. LKD had a significant risk of developing AKI of any degree after surgery compared with PN and RN. The difference was statistically significant using the Log-rank test (*p* > 0.001).

Based on the survival analysis, LKD patients were at a higher risk of developing AKI of every degree after 24 h from the procedure compared with other groups (LKD 79.4%, RN 7.4%, PN 5.6%). This difference decreased at discharge (LKD 84%, RN 71%, PN 29%). However, the differences between the three curves remained significant at the Log-rank test (*p* < 0.001)(Figure 2).

The one-way ANOVA confirmed this finding; however, the post hoc analysis clarified that LKD patients suffered exclusively from AKI stage I (100%, *p* = 0.028). Conversely, in PN patients, there was a significant prevalence of AKI stage II (*p* = 0.045), while in RN patients, every degree of AKI was represented almost evenly.

**Figure 3:**
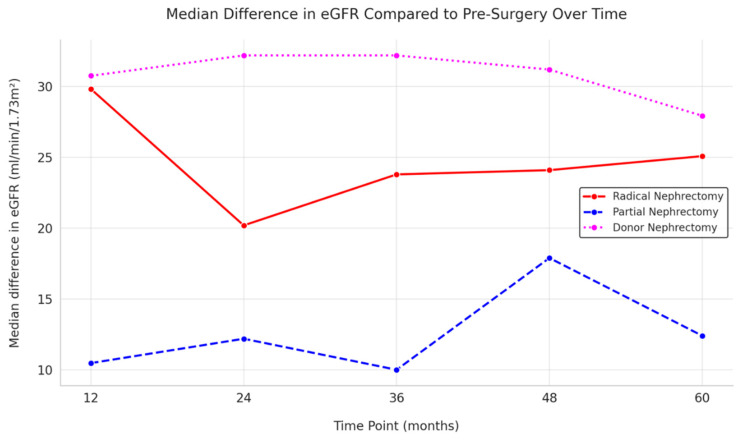
This line graph illustrates the median difference in eGFR (CKD-EPI) compared to pre-surgery values over time, stratified by the type of surgery, from 12 to 60 months after the surgery: radical nephrectomy is represented in red, partial nephrectomy is represented in blue, and donor nephrectomy is represented in violet.

After 12 months from surgery, the median difference of eGFR with pre-surgery was −29.83 mL/min/1.73 m^2^ (IQ 17.83) in the RN group, −10.48 mL/min/1.73 m^2^ (IQ 18.52) in the PN group, and −30.76 mL/min/1.73 m^2^ (IQ 15.72) in the LKD group. Sixty months after surgery, the median eGFR difference was −25.09 mL/min/1.73 m^2^ (IQ 19.34) in the RN group, −12.41 mL/min/1.73 m^2^ (IQ 20.76) in the PN group, and −27.94 mL/min/1.73 m^2^ (IQ 17.07) in the LKD group (Figure 3).

**Figure 4:**
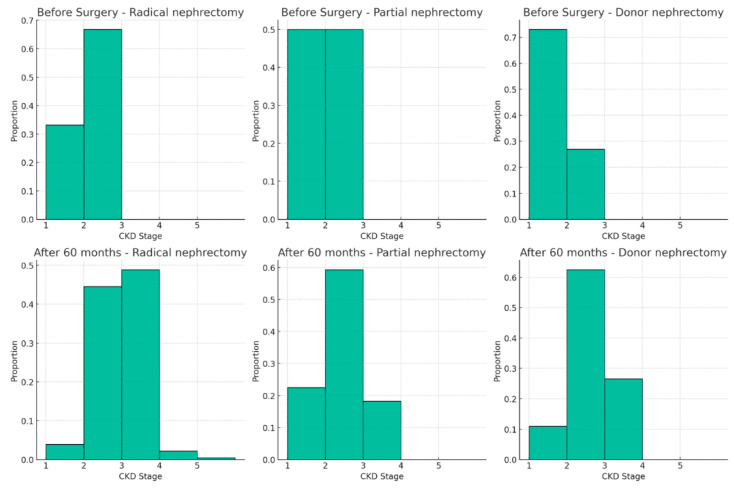
The plots depict the proportions of patients at each CKD stage before surgery (**top row**) and 60 months after surgery (**bottom row**) for each type of surgery.

Of note, the slope of kidney function between 12 and 60 months was indicative of a persistent compensatory effect in RN (0.10 mL/min/1.73 m^2^/year, IQ 3.94) and LKD (−0.08 mL/min/1.73 m^2^/year, IQ 2.35), while this effect was only partially observed in PN (−0.54 mL/min/1.73 m^2^/year). Translating these outcomes in CKD classes, after 60 months, the LKD group had a prevalence of CKD stage G3 (a + b) of 26.56%, while PN and RN were 18.22% and 48.91%, respectively; only a few RN patients experienced a severe decrease of kidney function (CKD stage G4 0.43%; G5 0.04%) (Figure 4).

RN showed the most pronounced renal function decline, with a −29.12% change in eGFR over 60 months compared to LKD (-26.95%), reflecting the complete loss of one kidney. PN resulted in a more moderate eGFR reduction of −15.17% due to nephron-sparing effects (Table 2).

Table 2 shows the mean eGFR values pre-surgery and after 60 months for living kidney donors (LKD), partial nephrectomy (PN), and radical nephrectomy (RN). The eGFR difference and percentage change indicate the long-term renal impact of each surgical type.

Among the considered continuous and categorical variables (age, sex, smoke, BMI, hypertension, diabetes, CKD pre-surgery, surgery type, and degree of AKI), we did not find an association with increased odds of developing AKI after surgery; on the other hand, age (OR: 1.07, 95% CI 1.044–1.092; *p* < 0.001), CKD pre-surgery (OR: 9.3, 95% CI 5.605–15.464; *p* < 0.001), the surgery type (*p* < 0.001), and any AKI event (OR 3.561, 95% CI 2.261–5.611; *p* < 0.001) were associated with increased odds to develop, in the long term, CKD stage progression (Table 3 and Table 4). Stratifying for surgery and comparing with the control group (LKD), RN was associated with a nonsignificant risk of CKD progression compared to LKD (*p* = 0.43). At the same time, PN showed a protective effect (OR: 0.326, 95% CI 0.168–0.632, *p* < 0.001).

## 4. Discussion

AKI and CKD are two significant sequelae of kidney surgery associated with both in-hospital and long-term mortality, increased risk of cardiovascular events, and progression to ESKD [14,15]. In the general population, proper treatment and medical follow-up are central to preventing severe kidney dysfunction post-surgery and during life. Our study investigated the incidence of perioperative AKI and CKD development over time in a cohort of 721 patients (465 affected by kidney cancer and 256 living donors) who underwent kidney surgery, either with radical and partial approach or donor nephrectomy. Our analyses were conducted to evaluate if the primum movens to conceive acute and chronic kidney dysfunction were related to surgery, baseline bio-humoral parameters, comorbidities, or all.

AKI development. Firstly, the incidence of perioperative AKI was higher in the two groups of nephrectomized patients (oncological and living donors) compared to the PN group. These results underline that kidney surgery had a higher impact in this group of patients compared to baseline bio-humoral parameters and pre-existing comorbidities. The acute loss of half nephron mass represented a functional shock in oncological and living donor cohorts regardless of the presence or absence of baseline diseases. On the contrary, when surgeons performed a conservative approach using PN, the preservation of the parenchyma of the affected kidney avoided the surgical trauma observed in RN/DN. Nonetheless, in the PN cohort, 29% of patients developed AKI, with a prevalence of AKI stage II. This result could be explained by a loss of functioning nephrons caused by several factors, such as excessive excision and reconstruction of kidney parenchyma and ischemia-reperfusion injury induced by clamping the hilar vessels.

Unexpectedly, living donors had a higher incidence of AKI than their oncological counterparts (LKD 84%, RN 71%, PN 29%). However, it is worth emphasizing that most AKIs in LKD are almost exclusively of stage I (100%). These results are hypothesis-generating. As discussed in a few papers, one possible explanation is that in the oncological RN cohort, the presence of cancer had already functionally compromised part of the parenchyma of the affected kidney so that the healthy counterpart had already started to vicariate the global function with a mild compensatory hemodynamic mechanism of hyperfiltration [2,16]. On the contrary, LKD had two healthy kidneys. Thus, the contralateral kidney is not “functionally prepared” when performing DN. This sudden “shock” can leave the residual kidney unable to compensate for the acute loss of kidney function.

Another possible interpretation of AKI in this subgroup may be linked with the definition of AKI itself. As previously discussed, the onset of AKI after nephrectomy is not an organic disease but an acute loss of nephron mass due to the surgery. Since LKD are healthy and unaffected by a parenchymal disease, developing prompt adaptive mechanisms results in a “surgically” derived AKI but of limited severity [17,18].

On the other hand, non-surgical AKI includes several clinical symptoms and signs due to the drop in GFR and the reduction of urinary output. These are not observed in surgical AKI, which is characterized by only an increase of serum creatinine 24-48 h after the intervention without any diuresis modification or electrolyte imbalance, as we observed in the LKD group. Therefore, the higher incidence of AKI in LKD could be interpreted as a “physiological” adaptation to a significant drop in kidney mass.

Finally, our logistic regression did not exhibit the expected statistical significance of specific covariates to predict AKI (e.g., diabetes, hypertension). Intriguingly, AKI, predominantly of stage I, was a frequent event among the LKD patients despite the low prevalence of diabetes (0.1%) and hypertension (16.0%) within our cohort. This commonality might have mitigated the anticipated differences between the surgical categories.

CKD development. Chronic kidney disease post-surgery trajectory exhibits variances across RN, PN, and LKD cohorts over 60 months. Patients who underwent an RN developed a CKD Stage 3 with a prevalence of 48.9% and Stage 4 at 0.43%. Conversely, the PN group exhibited an 18.22% incidence of Stage 3 CKD, while LKD participants encountered Stage 3 CKD at a rate of 26.56%.

This investigation delineates the impact of surgical intervention on kidney function, employing a tripartite analytical framework. Firstly, we analyzed the decline of eGFR from baseline pre-surgery to the end of observation at 60 months from the surgery. Findings indicated a mean eGFR reduction of −25.09 mL/min/1.73 m^2^ (IQR: 19.34) within the RN cohort, −12.41 mL/min/1.73 m^2^ (IQR: 20.76) for PN participants, and −27.94 mL/min/1.73 m^2^ (IQR: 17.07) among LKD subjects. In line with the reasons mentioned above for the development of AKI, our results confirmed that kidney surgery played a pivotal role in chronic kidney dysfunction over time, suggesting that complete kidney unit loss exerts a more pronounced effect on eGFR decline than pre-existing metabolic or vascular comorbidities. Furthermore, our analysis posits that surgically induced CKD represents an incomplete physiological adaptation by the remaining kidney to re-establish baseline kidney function. This adaptation may manifest as either effective compensatory hyperfiltration or a suboptimal response, with mean eGFR values at 60 months post-surgery evidencing this dynamic (69.3 ± 18.0 mL/min/1.73 m^2^).

Secondly, evaluating eGFR decrement from baseline to 12 months post-surgery, we pinpointed this interval as critical to developing the compensatory kidney adaptation, a process not as pronounced in PN subjects. Literature supports this period as a crucial “window” for compensatory kidney adjustments following unilateral nephrectomy, involving hemodynamic modifications, enhanced glomerular filtration, and augmented tubular reabsorption [19].

When we considered the eGFR modifications from 12 to 60 months, the slope of kidney function was indicative of a persistent compensatory effect in RN (0.10 mL/min/1.73 m^2^/year, IQ 3.94) and LKD (−0.08 mL/min/1.73 m^2^/year, IQ 2.35). This effect was only partially observed in PN (−0.54 mL/min/1.73 m^2^/year), who did not experience an actual relevant loss of kidney parenchyma and tended not to compensate with a trend directed as the general population.

Taking all these three different timelines, we can also speculate that, especially in LKD, the decrease of kidney function at 5 years from the surgery is not insidious or severe because the mean eGFR at the end of follow-up is 71.2 (±15.3) mL/min/1.73 m^2^, and therefore not significative to evolve in advanced stages of CKD. We consider this finding to be the most prominent one in the study. In fact, 12 months after the surgery, the LKD annual kidney function slope is lower than the general population, meaning that the remnant organ has started its hyperfiltration properly, protecting LKD from maladaptive mechanisms over time. Obviously, this can happen only if LKD are correctly selected pre-operatively and then followed with personalized nephrological counseling during their lifespan. The long-term risks of living donors are of utmost importance: Mjoen et al. in Norway and Muzaale et al. from the US each reported a small increased LKD ESKD risk in relative risk [20,21]. Nevertheless, a review by the ERA-EDTA-DESCARTES group points out that any increased risk must be taken in context with the actual risk in controls and is more relevant to the long-term “absolute risk”, which is close to zero [22]. Moreover, to date, the data suggests that any increased risk of ESKD is limited to first-degree relatives of the recipient. Additionally, since the role of new-onset diseases in LKD might impact their long-term outcome, it becomes mandatory to follow them closely post-donation and commit them to a healthy lifestyle and regular health maintenance check-ups that screen for early kidney disease.

Finally, our analysis highlighted that age (OR: 1.07, 95% CI 1.042–1.113; *p* < 0.0001), CKD pre-surgery (OR: 8.079, 95% CI 3.899–16.739; *p* < 0.0001), and the surgery type were associated with increased odds to develop, in the long term, CKD stage progression. These findings are in line with nephrological and urological literature.

This study, however, is not without limitations. Firstly, the GFR in our patients was not measured with iohexol/iothalamate plasma clearance but only estimated using the CKD-EPI formula. Therefore, eGFR could be occasionally overestimated, especially in oncological patients, due to a misleadingly low serum creatinine derived from reduced muscle masses. Secondly, in most of our patients, no kidney scintigraphy or other radiological techniques were performed after kidney surgery to highlight the changes in the remnant kidney and monitor the hyperfiltration mechanisms. Thirdly, no kidney histological evaluations were performed at the time of surgery to link the kidney function decay in acute and chronic scenarios from a specific baseline to glomerular, tubular, vascular, or interstitial dysfunction. Fourthly, although ischemic time during PN can significantly affect renal outcomes due to ischemia-reperfusion injury, we did not have comprehensive data on the ischemia time in this group.

## 5. Conclusions

This study highlights several important considerations for the management of both kidney cancer (KC) patients undergoing partial or radical nephrectomy and living kidney donors (LKD), with a particular focus on the risks of acute kidney injury (AKI) and chronic kidney disease (CKD). Our work, indeed, contributes new insights by demonstrating that the rate of kidney function deterioration, both in the acute and chronic settings, is similar between KC patients and LKD individuals. This finding suggests that the type of surgical intervention—whether partial nephrectomy (PN), radical nephrectomy (RN), or kidney donation—plays a more decisive role in determining post-operative AKI and CKD incidence than underlying patient characteristics such as the presence or absence of malignancy. Therefore, our work suggest a possible reevaluation of current guidelines for managing patients with a solitary kidney, with the goal of enhancing long-term renal status in both kidney donors and cancer patients

Future studies should aim to further explore the mechanisms underlying these similarities in renal outcomes between the two groups and assess how different surgical techniques can be optimized to reduce the risk of post-operative renal complications.

## Figures and Tables

**Table 1:** Descriptive statistics of the cohort. Radical nephrectomy (RN); partial nephrectomy (PN); donor nephrectomy (LKD); standard deviation (SD).

Variables	Total	RN	PN	RN + PN	LKD
Patients—no. (%)	721	229 (31.8%)	236 (32.7%)	465 (64.4%)	256 (35%)
Age—years					
Mean	54.97	56.90	57.18	57.04	50.8
±SD	10.91	10.83	10.74	10.78	9.94
Male sex—no. (%)	392 (54.4)	163 (71.2)	156 (66.1)	319 (68.6)	73 (28.5)
BMI (kg/m^2^)					
Mean	25.6	26.5	25.83	26.16	24.7
±SD	4.13	4.81	4.20	4.50	3.25
Hypertension—no. (%)	232 (32.1)	93 (40.6)	98 (41.5)	191 (41.0)	41 (16.0%)
Diabetes—no. (%)	34 (4.7)	14 (6.1)	19 (8.0)	33 (7.1)	1 (0.1)
Smoke—%	37.4	47.6	38.8	43.2	33.74
Serum Creatinine (mg/dL)—Basal					
Mean	0.84	0.89	0.84	0.86	0.78
±SD	0.16	0.15	0.16	0.15	0.14
CKD-EPI (mL/min/1.73 m^2^)—Basal					
Mean	91.3	87.2	91.1	89.1	97.40
±SD	13.42	11.6	12.5	12.0	13.27

**Table 2:** Mean eGFR at baseline and after 60 months.

Surgery Type	Mean eGFR Pre-Surgery	Mean eGFR After 60 Months	Mean eGFR Difference	% Change
**LKD**	97.52	71.24	−26.28	−26.95
**PN**	90.15	76.48	−13.68	−15.17
**RN**	85.8	60.81	−24.99	−29.12

**Table 3:** Logistic univariate analysis predicting AKI onset after surgery. OR, odds ratio; C.I. confidence interval; CKD, chronic kidney disease; LKD, living kidney donor nephrectomy; RN, radical nephrectomy; PN, partial nephrectomy; BMI, body mass index.

			Univariate			
Covariates			*p* Value	OR	95% C.I. for OR	
					Lower	Upper
	**Male vs. Female**		0.510	0.625	0.154	2.535
	**Age (years)**		0.570	1.021	0.950	1.097
	**CKD pre-surgery (Y/N)**		0.674	0.693	0.125	3.842
	**LKD vs. RN vs. PN**	**LKD**	0.917			
		**RN**	0.690	0.676	0.099	4.633
		**PN**	0.912	1.130	0.130	9.823
	**Smoke (Y/N)**		0.652	0.740	0.200	2.739
	**BMI (kg/m^2^)**		0.215	1.110	0.941	1.311
	**Hypertension (Y/N)**		0.701	0.750	0.172	3.257
	**Diabetes (Y/N)**		0.792	0.701	0.050	9.855

**Table 4:** Logistic univariate and multivariate analysis of independent variables with the outcome (CKD stage progression). OR, odds ratio; C.I. confidence interval; CKD, chronic kidney disease; LKD, living kidney donor; RN, radical nephrectomy; PN, partial nephrectomy; AKI, acute kidney injury.

		Univariate				Multivariate			
Male vs. Female		*p* Value	OR	95% C.I. for OR		Sig.	OR	95% C.I. for OR	
				Lower	Upper			Lower	Upper
**Sex (male)**		0.075	0.733	0.521	1.032				
**Age (year)**		0.082	1.014	0.998	1.030	**<0.001**	1.068	1.044	1.092
**Smoke (Y/N)**		0.577	1.154	0.698	1.907				
**BMI (kg/m^2^)**		0.749	0.993	0.955	1.034				
**Hypertension (Y/N)**		0.330	0.843	0.598	1.189				
**Diabetes (Y/N)**		0.832	1.082	0.522	2.245				
**CKD pre-surgery (Y/N)**		**<0.001**	3.792	2.656	5.414	**<0.001**	9.310	5.605	15.464
**LKD vs. RN vs. PN**	**LKD**	**<0.001**				**<0.001**			
	**RN**	0.079	0.633	0.380	1.055	0.437	1.295	0.674	2.487
	**PN**	**<0.001**	0.214	0.130	0.352	**<0.001**	0.326	0.168	0.632
**AKI (Y/N)**		**<0.001**	4.280	2.996	6.116	**<0.001**	3.561	2.261	5.611
**AKI severe (II–III) (Y/N)**		0.426	1.567	0.519	4.731				

## Data Availability

Research data are unavailable due to privacy or ethical restrictions.

## References

[1] Capitanio U., Montorsi F. (2016). Renal cancer. Lancet.

[2] Di Marco F., Pani A., Floris M., Martini A., Dell’Antonio G., Capitanio U., Bettiga A., Larcher A., Cinque A., Bertini R. (2021). Unexpected Outcomes of Renal Function after Radical Nephrectomy: Histology Relevance along with Clinical Aspects. J. Clin. Med..

[3] van der Weijden J., Mahesh S.V.K., van Londen M., Bakker S.J.L., Sanders J.-S., Navis G., Pol R.A., Roodnat J.I., Kho M.M.L., Yakar D. (2022). Early increase in single-kidney glomerular filtration rate after living kidney donation predicts long-term kidney function. Kidney Int..

[4] Tantisattamo E., Dafoe D.C., Reddy U.G., Ichii H., Rhee C.M., Streja E., Landman J., Kalantar-Zadeh K. (2019). Current Management of Patients With Acquired Solitary Kidney. Kidney Int. Rep..

[5] Shrivastava N., Sharma G., Ahluwalia P., Gautam G., Erdem S., Amparore D., Marchioni M., Pavan N., Marandino L., Roussel E. (2023). Off-clamp Versus On-clamp Robot-assisted Partial Nephrectomy: A Systematic Review and Quantitative Synthesis by the European Association of Urology Young Academic Urologists Renal Cancer Study Group. Eur. Urol. Open Sci..

[6] Thompson R.H., Lane B.R., Lohse C.M., Leibovich B.C., Fergany A., Frank I., Gill I.S., Blute M.L., Campbell S.C. (2010). Every minute counts when the renal hilum is clamped during partial nephrectomy. Eur. Urol..

[7] Mir M.C., Derweesh I., Porpiglia F., Zargar H., Mottrie A., Autorino R. (2017). Partial Nephrectomy Versus Radical Nephrectomy for Clinical T1b and T2 Renal Tumors: A Systematic Review and Meta-analysis of Comparative Studies. Eur. Urol..

[8] Park J.J., Kim K., Choi J.Y., Shim S.R., Kim J.H. (2021). Long-term mortality of living kidney donors: A systematic review and meta-analysis. Int. Urol. Nephrol..

[9] Park J.Y., Yang W.J., Doo S.W., Park J.J., Gwon Y.N., Kim K.M., Kim J.H., Kim D.K. (2023). Long-term end-stage renal disease risks after living kidney donation: A systematic review and meta-analysis. BMC Nephrol..

[10] Locke J.E., Reed R.D., Massie A.B., MacLennan P.A., Sawinski D., Kumar V., Snyder J.J., Carter A.J., Shelton B.A., Mustian M.N. (2019). Obesity and long-term mortality risk among living kidney donors. Surgery.

[11] Lee S.H., Kim D.S., Cho S., Kim S.J., Kang S.H., Park J., Park S.Y., Chang S.-G., Jeon S.H. (2015). Comparison of postoperative estimated glomerular filtration rate between kidney donors and radical nephrectomy patients, and risk factors for postoperative chronic kidney disease. Int. J. Urol. Off. J. Jpn. Urol. Assoc..

[12] Gazel E., Biçer S., Ölçücüoğlu E., Yığman M., Taştemur S., Çamtosun A., Ceylan C., Ateş C. (2015). Comparison of renal function after donor and radical nephrectomy. Ren. Fail..

[13] Khwaja A. (2012). KDIGO clinical practice guidelines for acute kidney injury. Nephron Clin. Pract..

[14] Wensong W., Fan C., Jianghui Z., Shuai T., Zheng L., Xuehui L., Fangmin C. (2023). Correlation between bilateral GFR in patients with localized renal cancer after partial nephrectomy. Int. Urol. Nephrol..

[15] Capitanio U., Terrone C., Antonelli A., Minervini A., Volpe A., Furlan M., Matloob R., Regis F., Fiori C., Porpiglia F. (2015). Nephron-sparing techniques independently decrease the risk of cardiovascular events relative to radical nephrectomy in patients with a T1a-T1b renal mass and normal preoperative renal function. Eur. Urol..

[16] Patel H.D., Pierorazio P.M., Johnson M.H., Sharma R., Iyoha E., Allaf M.E., Bass E.B., Sozio S.M. (2017). Renal Functional Outcomes after Surgery, Ablation, and Active Surveillance of Localized Renal Tumors: A Systematic Review and Meta-Analysis. Clin. J. Am. Soc. Nephrol. CJASN.

[17] Yang X., Zhang T., Zhou H., Ni Z., Wang Q., Wu J., Chen Q., Qiu M., Wang Y., Fu T. (2023). Acute kidney injury as an independent predicting factor for stage 3 or higher chronic kidney disease after nephrectomy. Urol. Oncol..

[18] Acute Kidney Injury (AKI)—KDIGO. https://kdigo.org/guidelines/acute-kidney-injury/.

[19] Hew M.N., Opondo D., Cordeiro E.R., van Donselaar-van der Pant K.A.M.I., Bemelman F.J., Idu M.M., de la Rosette J.J.M.C.H., Laguna M.P. (2014). The 1-year decline in estimated glomerular filtration rate (eGFR) after radical nephrectomy in patients with renal masses and matched living kidney donors is the same. BJU Int..

[20] Mjøen G., Hallan S., Hartmann A., Foss A., Midtvedt K., Øyen O., Reisæter A., Pfeffer P., Jenssen T., Leivestad T. (2014). Long-term risks for kidney donors. Kidney Int..

[21] Muzaale A.D., Massie A.B., Wang M.-C., Montgomery R.A., McBride M.A., Wainright J.L., Segev D.L. (2014). Risk of end-stage renal disease following live kidney donation. JAMA.

[22] Grossi A.A., Sever M.S., Hellemans R., Mariat C., Crespo M., Watschinger B., Peruzzi L., Demir E., Velioglu A., Gandolfini I. (2023). The 3-Step Model of informed consent for living kidney donation: A proposal on behalf of the DESCaRTES Working Group of the European Renal Association. Nephrol. Dial. Transplant. Off. Publ. Eur. Dial. Transpl. Assoc.—Eur. Ren. Assoc..

